# Androgen dihydrotestosterone promotes bladder cancer cell proliferation and invasion via EPPK1-mediated MAPK/JUP signalling

**DOI:** 10.1038/s41419-023-05882-1

**Published:** 2023-06-16

**Authors:** Long Yang, Wen Huang, Xiaoyu Bai, Haoyu Wang, Xiaolei Wang, Huiyuan Xiao, Yanlei Li

**Affiliations:** 1 Department of Urology, Tianjin Medical University General Hospital, Tianjin, China; 2 Department of Pathology, Tianjin Medical University Cancer Institute & Hospital, Tianjin, China; 3 Department of Pathology, Tianjin Medical University, Tianjin, China

**Keywords:** Tumour biomarkers, Cell death

## Abstract

The incidence of bladder cancer (BLCA) in men is higher than that in women. Differences in androgen levels between men and women are considered the main causes of incidence rate differences. In this study, dihydrotestosterone (DHT) significantly increased the proliferation and invasion of BLCA cells. In addition, BLCA formation and metastatic rates were higher in N-butyl-N-(4-hydroxybutyl) nitrosamine (BBN)-treated male mice than in female and castrated male mice in vivo. However, immunohistochemistry showed that androgen receptor (AR) was expressed at low levels in normal and BLCA tissues of men and women. The classical AR pathway considers that DHT binds to AR and induces it to enter the nucleus, where it functions as a transcription factor. Here, a non-AR combination pathway of androgen that promoted BLCA development was investigated. The EPPK1 protein was bombarded with DHT, as determined by biotinylated DHT-binding pull-down experiments. EPPK1 was highly expressed in BLCA tissues, and EPPK1 knockdown significantly inhibited BLCA cell proliferation and invasion promoted by DHT. Moreover, JUP expression was elevated in DHT-treated high-EPPK1 expressing cells, and JUP knockdown inhibited cell proliferation and invasion. EPPK1 overexpression increased tumour growth and JUP expression in nude mice. Furthermore, DHT increased the expression of the MAPK signals p38, p-p38, and c-Jun, and c-Jun could bind to the JUP promoter. However, the promotion of p38, p-p38, and c-Jun expression by DHT was not observed in EPPK1 knockdown cells, and a p38 inhibitor suppressed the DHT-induced effects, indicating that p38 MAPK may be involved in the regulation of DHT-dependent EPPK1-JUP-promoted BLCA cell proliferation and invasion. The growth of bladder tumours in BBN-treated mice was inhibited by the addition of the hormone inhibitor goserelin. Our findings indicated the potential oncogenic role and mechanism of DHT in BLCA pathogenesis through a non-AR pathway, which may serve as a novel therapeutic target for BLCA.

## Introduction

Bladder cancer (BLCA) represents a common genitourinary malignancy worldwide and causes a high mortality rate in patients with urinary tract disease, with approximately 165,100 deaths annually [1]. Excessive exposure of men to cigarette smoke and industrial chemicals, both of which include amines, could result in the development of BLCA [2]. However, sex-related differences in the risk of BLCA can persist in the absence of exposure to known carcinogenic factors. Males typically have a threefold higher risk of being diagnosed with BLCA than females [3], but the reasons for such sex-related differences in the risk of BLCA remain unclear [4]. Based on previous reports, androgens may play a role in regulating bladder urothelial tumourigenesis, which may explain its higher risk in men [5]. Others have also shown that androgens increase cell proliferation in vitro and in vivo, and anti-androgen treatment inhibits the effect of androgens [6, 7]. Previous studies have reported that postmenopausal women also have a higher incidence of BLCA than premenopausal women [8], which also indicates the potential role of androgens in BLCA development. Thus, the basis for the sex-specific difference in BLCA incidence remains unknown.

DHT is generated by the 5α-reduction of testosterone, and it cannot be aromatised to oestradiol. Therefore, it is considered a pure androgenic steroid. A potential mediator of sex-specific differences is AR, which is a member of the nuclear receptor superfamily, and it is a ligand-dependent transcription factor that mediates the biological effect of androgens [9]. AR expression has been detected in the normal bladder epithelium [10] and BLCA in male and female patients. However, studies on AR function in the bladder or androgen metabolism in the bladder urothelium are limited. In addition, no consensus on the role of AR in BLCA has been found because some studies are contradictory [11, 12].

Epiplakin1 (Eppk1), a member of the plakin gene family, is a universal cell junction protein that has been identified as a human epidermal autoantigen, which is ubiquitously expressed in the oesophagus and several other organs [13]. It is a common cell junction protein and part of EGF signalling, which plays a role in cytoskeletal aggregation and proliferation signalling in tumour cells [14]. Different expression levels of Eppk1 have been found in various cancers, such as hepatocellular carcinoma [15], cervical cancer [16], and bladder urothelial carcinoma [17], but the role of Eppk1 in BLCA remains unknown.

JUP, also referred to as γ-catenin and plakoglobin, is a structural and functional homologue of β-catenin and Armadillo, both of which play adhesion and signalling roles in development [18]. JUP is an adhesion protein; thus, insufficient expression of JUP reduces cell–cell contact and increases the invasion and spread of cancer cells in the body [19]. In some tumour entities, the loss of JUP expression results in adverse tumour characteristics, which are associated with increased tumour stage, poor patient survival, and increased metastasis [20, 21]. However, JUP is overexpressed in some malignancies, including breast cancer [22], acute myeloid leukaemia [23], and lung adenocarcinoma [24]. Therefore, whether JUP serves as a tumour suppressor or an oncogenic protein in BLCA is unknown.

At present, the effect and mechanism of DHT on BLCA among male and female patients remain unknown. In the present study, DHT may not play a role in BLCA progression through AR. Subsequent studies have shown that DHT can interact with EPPK1 to promote the expression of JUP through the P38 MAPK/c-JUN signalling pathway, leading to the aggressive progression of BLCA. Therefore, our findings can facilitate the development of effective therapeutic strategies for BLCA.

## Material and methods

### Patients

All 619 BLCA patients’ clinicopathological characteristics were collected from patients undergoing surgical resection at the General Hospital of Tianjin Medical University from 2016 to 2018. The patients included 425 men and 194 women. Pairs of 60 BLCA tissues and adjacent normal bladder tissues were obtained from BLCA patients for immunohistochemistry. Six cases of fresh BLCA tissues taken from surgical resection from the Department of Urology, General Hospital of Tianjin Medical University, were used for qRT-PCR. Six males and six females’ paraffin-embedded tissue from the Department of Pathology, General Hospital of Tianjin Medical University, were used for DNA sequencing. This study was approved by the Ethics Committee of the General Hospital of Tianjin Medical University, and all protocols conformed to the Ethical Guidelines of the World Medical Association Declaration of Helsinki. Signed informed consent was obtained from each participating individual prior to participation in the study.

### Immunohistochemistry

Formalin-fixed tissues were embedded in paraffin, deparaffinized and rehydrated according to standard procedures for immunohistochemistry. Deparaffinized tissue slides were blocked with 3% H_2_O_2_ solution, and antigen was recovered with 10 mM citrate buffer (pH = 6.0). After 30 min of blocking, appropriately diluted primary antibodies were added to slides and incubated overnight in a humidified chamber at 4 °C, and then appropriately diluted biotinylated secondary antibodies were added at room temperature. After 1 h of incubation, DAB was used to develop colour. Finally, nuclei were localised by haematoxylin staining.

The immunohistochemical staining results were assigned a score considering both the intensity of the staining and the proportion of tumour cells with an unequivocal positive reaction. Positive reactions were defined as those showing brown signals in the cell. A staining index (values, 0–12) was determined by multiplying the score of the staining intensity by the score of the positive area. The intensity was scored as follows: 0 (negative), 1 (weak), 2 (moderate), and 3 (strong). The proportion of positive cells was defined as follows: 0 (less than 5%); 1 (5–25%); 2 (26–50%); 3 (51–75%); and 4 (greater than 75%). In the statistical analysis, scores of 0 to 2 were considered negative or low expression, and scores of 3 to 12 were considered positive or high expression.

### Immunofluorescence (IF) staining

Cells were seeded on glass slides, and fixed with cold methanol for 15 min, then washed three times with PBS. Permeabilize with 1% Triton for an additional 30 min, wash three times with PBS, and block with 5% FBS for 30 min. Slides were incubated with primary antibodies overnight at 4 °C. The next day, the slides were rewarmed at room temperature for 1 h, stained with FITC-labelled second antibody, and incubated with the slides at 37 °C for 2 h in the dark. After counterstaining with DAPI, images were acquired using a Nikon Eclipse TS100 microscope.

Antibodies used included primary antibodies against AR (ab108341), EPPK1 (ab247172), JUP (ab184919), c-JUN ((ab40766), p-P38 (ab178867), p38 (ab170099), and GAPDH (1:1000, Santa Cruz Biotechnology).

### Cell lines

The bladder cancer cell lines T24, 5637, J28, and UMUC3 were purchased from Yuchicell (Shanghai) Biological Technology Co., Ltd. All human cell lines have been authenticated using STR profiling. Foetal bovine serum (FBS) was purchased from Invitrogen. DMEM was obtained from Invitrogen. All cell lines were cultured in DMEM supplemented with 10% foetal bovine serum (FBS) at 37 °C and 5% CO_2_ in an incubator.

### Plasmids and transfection

ShRNA and negative control plasmids for AR, EPPK1 and JUP were constructed by GeneCopoeia (Guangzhou, China) and used to transfect T24 cells with Lipofectamine 2000 (Invitrogen, USA). Puromycin (Sigma, USA) was used to screen stably transfected cells.

### Cell proliferation assay

A total of 5 × 10^4^ cells/well were cultured overnight in 6-well plates. After 24 h of growth, the experimental test cells were treated with DHT and clonal cells were identified as those capable of forming colonies consisting of at least 50 cells. Colonies were fixed with methanol, stained with 0.5% crystal violet and photographed.

### Invasion assay

The cells were digested, a serum-free cell suspension was prepared, the number of cells was adjusted to 5 × 10^4^ cells/mL, and 20 μL Matrigel gel was placed in the Transwell chamber for the invasion experiment. Then, 500 μL of complete medium was added to the upper chamber of the 24-well plate and 200 μL of the serum-free cell suspension to the lower chamber. After 48 h in the invasion experiment, cells were fixed with cold methanol for 20 min and stained with crystal violet for 30 min. Five visual fields in the chamber were randomly selected and imaged using a Nikon Eclipse TS100 microscope. The assay was independently performed in triplicate.

### Enzyme-linked immunosorbent assay (ELISA)

The collected mouse eye blood was centrifuged to collect serum. Blank control, negative control, and 100 microlitres of samples were added to the reaction wells of each titre plate and incubated at 37 °C for 1 h. After washing three times, 100 μL of enzyme-labelled antibody to each reaction well and incubate at 37 °C for 30 min. After washing three times, colour developing solution was added to each reaction well, and the OD value was measured at 450 nm with a microplate reader.

### Western blot analysis

Protein was extracted from the cell lysate, and electrophoresis was performed. The proteins were transferred to a PVDF membrane for 90 min, blocked in 5% skimmed milk powder for 1 h, added to the primary antibody, and incubated on a shaker at room temperature for 1 h and overnight at 4 °C. On the second day, the corresponding secondary antibody was added and incubated at room temperature for 2 h. The grey value of the protein bands was analysed with ImageJ. Primary antibodies against AR, JUP and GAPDH were used according to the manufacturers’ guidelines. Rabbit or mouse HRP-conjugated secondary antibodies diluted with antibody diluent at a ratio of 1:1000 was purchased from Santa Cruz Biotechnology (USA).

### Pull-down assay

Biotin-labelled DHT was synthesised by HUA Yen Biotech. Then, 10^7^cells were washed in ice-cold phosphate-buffered saline, lysed in 500 μL Co-IP buffer (20 mM Tris-HCL, pH 7.5, 150 mM NaCl, 1 mM EDTA, 0.5% NP-40, and complete protease inhibitor cocktail and RNase inhibitors), and incubated with 3 μg biotinylated DNA oligo probes, at room temperature for 2 h. A total of 50 μL washed streptavidin C1 magnetic beads (Invitrogen) were added to each binding reaction and further incubated at room temperature for another hour. The beads were washed briefly with Co-IP buffer five times. The bound proteins in the pull-down materials were analysed by mass spectrometry and western blotting.

### Mass spectrometry

The bound protein-digested sample was injected into a Nano-LC system (EASY-nLC 1200, Thermo Fisher Scientific). Each sample was separated by a C18 column (50-μm inner-diameter × 15 cm, 2 μm C18) at a flow rate of 300 nL/min. The HPLC gradient was as follows: 5–13% solvent B (0.1% formic acid in acetonitrile) in 16 min, 13–28% solvent B in 35 min, 28–40% solvent B in 15 min, 40–100% solvent B in 1 min and hold for 8 min at 100% solvent B. The HPLC eluate was electrosprayed directly into an Orbitrap QE plus mass spectrometer (Thermo Fisher Scientific). The mass spectrometric analysis was carried out in a data-dependent mode. For the MS1 survey scan, the automatic gain control (AGC) target is 3e6 and the resolution is 70,000. The MS2 spectra were acquired with 17500 resolutions. Mass spectrometry analysis was performed by the Protein mass spectrometry analysis platform of Tianjin Medical University.

### Quantitative real-time polymerase chain reaction (qRT‒PCR)

Total RNA was extracted from bladder cancer tissues and paired with adjacent normal bladder tissues using TRIzol reagent. Two micrograms of total RNA were reverse-transcribed into cDNA using a reverse transcription kit (Takara, RR037A). Real-time PCR analysis was performed using Power SYBR Green (Takara, RR820A). All experiments were performed according to the manufacturer’s instructions. GAPDH was used as an endogenous control.

### DNA and RNA sequencing

Paraffin-embedded BLCA tissues were collected for DNA extraction. Tissue samples were lysed by proteinase K to release DNA. DNA was extracted by nucleic acid extraction reagent (model: FFPE DNA, Amoy Diagnostics Co., Ltd). The mutant of EPPK1 sequencing was detected by CTI (Centre Testing International Group) Medical Laboratories Co. Ltd. The mutant of EPPK1 was compared with the human reference genome GRCh37.

T24 cells treated with DHT were used for RNA sequencing. Total RNA was extracted from the cells using TRIzol reagent (Invitrogen). RNA sequencing analysis based on RNA quality and quantity, which were evaluated using a Bioanalyzer (Agilent) and Nanodrop (Thermo Fisher Scientific). All sequence data were assessed for quality control using FastQC/MultiQC [25, 26] and RseQC package [27]. RNA-seq was performed on an Illumina HiSeq^TM^ 2500 platform and the differentially expressed genes were analysed by CTI (Centre Testing International Group) Medical Laboratories Co. Ltd. (Shanghai, China) using DEGseq.

### ChIP

The chromatin fragments were reduced to 100–500 bp by sonication on ice for two cycles of 20 cycles. The obtained supernatant was divided into the IP group, IgG group, and input group, and immunoprecipitated with 5 μg of antibody at 4 °C. Chromatin was precleared for 3 h each with protein G agarose beads after immunoprecipitation. The immunoprecipitated material was washed, four times in RIPA buffer, two times in LiCl buffer and three times in TE buffer before elution in elution buffer. After the beads were removed, samples were cross-linked overnight at 65 °C. The antibodies used included primary antibodies against c-JUN and IgG. The target gene of each sample was subjected to qPCR. The PCR products were analysed using electrophoresis on a 2% agarose gel and were photographed after ethidium bromide staining.

### Luciferase reporter assay

For promoter reporter assays, the same cell numbers will be seeded in 6-well plates. Control reporter plasmid, c-JUN, JUP, and mutant JUP were cotransfected alone or combined into 293T cells; the JUP and mutant JUP reporter plasmids were cotransfected with c-JUN into DHT-treated T24 cells and their control cells, or the JUP reporter plasmid was co-transfected with c-JUN into DHT-treated EPPK1-knockdown T24 cells, pcDNA3-EPPK1 and its control cells. After 48 h, luciferase activity identification was performed by using a luciferase reporter gene kit.

### Animal experiments

Animal studies were approved by the Committee on Animal Research and Ethics of Tianjin Medical University, and all protocols conformed to the Guidelines for Ethical Conduct in the Care and Use of Nonhuman Animals in Research. First, we purchased littermates (male: *n* = 40; female: *n* = 20, all 5–6 weeks old). They were supplied ad libitum with tap water containing 0.05% BBN (Aladdin) in opaque bottles. The drinking water was prepared fresh twice a week, and consumption was recorded to estimate BBN intake. Male mice were randomly divided into two groups, that received surgical castration (*n* = 20) or sham surgery (*n* = 20) at 5 weeks of age before starting BBN treatment. After 25 weeks of feeding, mice were sacrificed and bled, and the tumour formation rate, metastasis rate, and testosterone concentration determination of mice were calculated. No data were excluded from our analyses. The investigator was blinded to the group allocation of the animals during the experiment. Immunohistochemical staining for AR, EPPK1 and JUP in metastatic tumour tissue.

For in vivo drug studies, mice were randomly divided into an experimental group and a control group. Each group was fed BBN, and the mice in the experimental group were intraperitoneally injected with goserelin at a dose of 50 mg/kg twice a week. After 25 weeks of feeding, mice were sacrificed and bled, and the tumour formation rate, metastasis rate, and testosterone concentration determination of mice were calculated.

In addition, 5 × 10^6^ T24 and T24-shEPPK1 cells were injected subcutaneously into 4-week-old BALB/c nude mice. Tumour size was measured weekly with Vernier callipers 1 week after inoculation. Tumour volumes were calculated as follows: volume (mm^3^) = (length [mm] ×width^2^ [mm])/2.

### Statistical analysis

All cell experiments were performed in triplicate. SPSS 25.0 was used to perform statistical analysis on the obtained data. Data are shown as the mean ± SD for at least three independent experiments. A two-sided Student’s *t*-test was performed to determine differences between the two groups of results, and two-way ANOVA was used for the comparison of more than two groups when the variance is similar between the groups. Kaplan‒Meier survival analysis was used for survival analysis. *p* < 0.05 was considered statistically significant.

## Results

### DHT promotes BLCA cell proliferation, invasion, and tumour formation

**Fig. 1:**
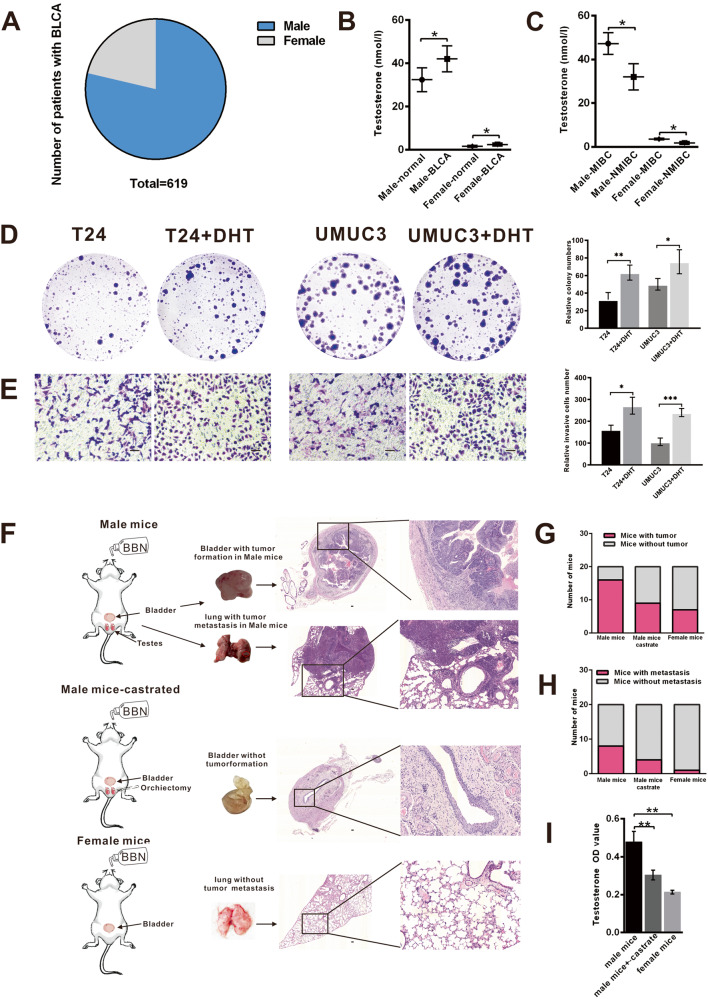
Function of DHT in bladder cancer. **A** The incidence rate of bladder cancer in men is three times that in women. **B** Testosterone concentrations in male and female BLCA patients were higher than those in normal men and women. **C** The testosterone level of patients with muscle-invasive bladder cancer (MIBC) was higher than that of patients with nonmuscle-invasive bladder cancer (NMIBC). **D** Colony formation assays showed that T24 and UMUC3 cell proliferation abilities were increased after adding DHT. **E** The invasion capacity of T24 and UMUC3 cells was increased after adding DHT, as assessed by Transwell assays. **F**–**H** Male mice after 25 weeks of BBN feeding showed more tumour formation and metastasis than castrated male mice and female mice. **I** Testosterone concentrations in male mice, castrated male mice, and female mice. Scale bar = 100 μm. *p < 0.05, **p < 0.01, ***p < 0.001.

Through the analysis of clinical patient data, it was confirmed that the incidence rate of bladder cancer in men was three times that in women (Fig. 1A). Testosterone concentrations in male and female BLCA patients were higher than those in normal men and women (Fig. 1B). Furthermore, the testosterone level of patients with muscle-invasive bladder cancer (MIBC) was higher than that of patients with nonmuscle-invasive bladder cancer (NMIBC) (Fig. 1C). We determined the role of DHT in BLCA cell lines and harvested mouse tumours using proliferation, invasion, and haematoxylin and eosin (HE) staining assays to explore the effects of DHT on BLCA development. As shown in Fig. 1D, E, compared with the control group, DHT addition in T24 and UMUC3 cells increased cell proliferation and invasion. In addition, we studied BLCA development in 60 C57 mice, which were divided into three groups: male mice, male castrated mice, and female mice. All three groups of mice were treated with BBN. After 30 weeks, bladder carcinoma was found in 80% of BBN-treated male mice, whereas only 35% of BBN-treated female mice had developed bladder tumours, and 45% of BBN-treated castrated male mice had developed bladder tumours (Fig. 1F, G). Eight mice in the male group developed metastatic tumours, whereas only three castrated male mice and one female mouse treated with BBN developed metastatic tumours (Fig. 1H). Using ELISA to measure serum testosterone concentration in mice, we found that male mice had the highest testosterone content, whereas female mice have the lowest testosterone content, and the testosterone content of castrated mice was lower than that of male mice (Fig. 1I). These results not only confirm that DHT promotes BLCA proliferation and metastasis but also indicate sex differences in BBN-induced BLCA incidence.

### DHT promotes BLCA progression not through AR signalling

**Fig. 2:**
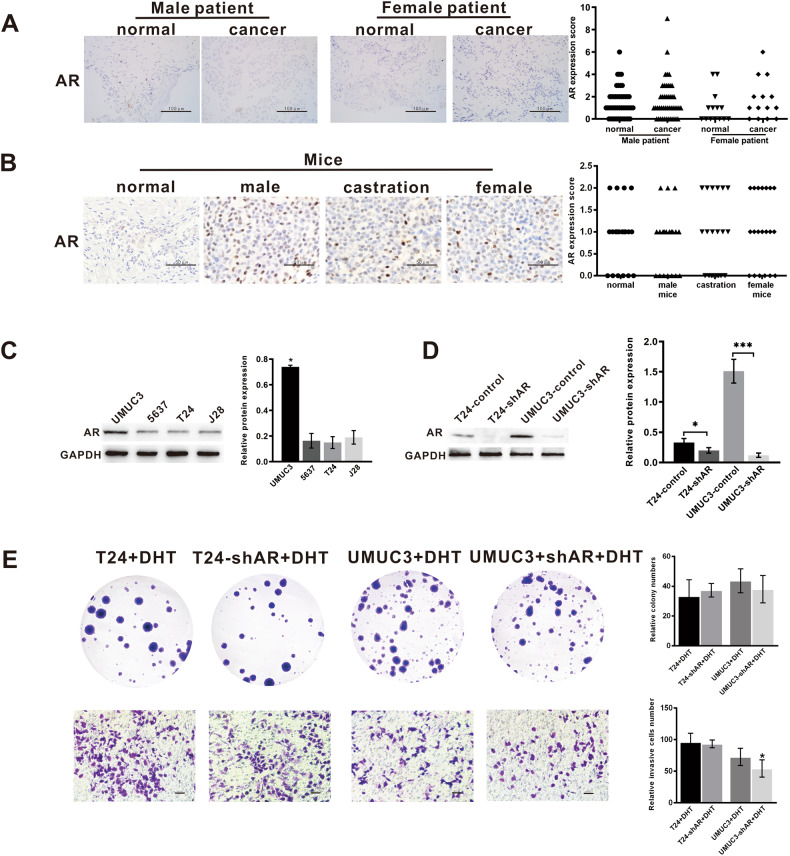
AR expression in bladder cancer. **A** AR was expressed at low levels in both cancer and normal tissues of males and females. **B** AR was expressed at low levels in normal mice, male mice, castrated male mice, and female mice. **C** AR expression in four BLCA cell lines. **D** Effect of downregulation of AR analysed by WB. **E** The proliferation and invasion abilities were decreased in T24-shAR+DHT and UMUC3-shAR+DHT cells compared with T24+DHT and UMUC3+DHT cells. For immunohistochemistry results, scores of 0 to 2 were considered negative or low expression, and scores of 3 to 12 were considered positive or high expression. Scale bar = 100 μm. *p < 0.05, ***p < 0.001.

To confirm whether testosterone plays a regulatory role through the AR signalling pathway in BLCA, we examined AR expression in tissues and cell lines by IHC and Western blotting (WB). AR detected by IHC showed low expression in 60 cases of human BLCA and their adjacent normal tissues and 60 mouse tumour tissues, and no sex difference was found (Fig. 2A, B). The expression levels of AR were detected in T24, UMUC3, 5637, and J28 cell lines, and AR expression was higher in UMUC3 cells and lower in T24, 5637, and J28 cells. AR was knocked down in T24 and UMUC3 cell lines and plasmid transfection success was detected by WB (Fig. 2C). Compared with the control group, the proliferation and invasion capacity of T24-AR knockdown and UMUC3-AR knockdown cells treated with DHT did not change significantly (Fig. 2D). Therefore, our study showed that AR had no sex difference in the incidence of BLCA, and DHT may have an effect on BLCA proliferation and invasion, but not through AR signalling.

### DHT interacts with the EPPK1 protein in BLCA

**Fig. 3:**
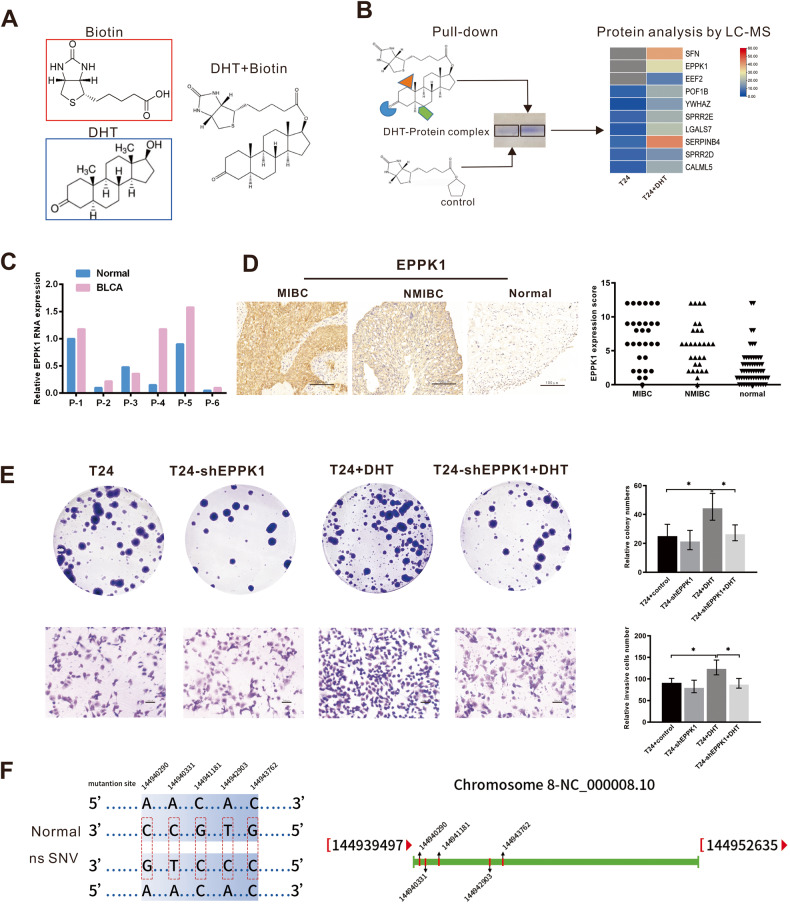
DHT interacts with the EPPK1 protein in bladder cancer. **A** Structural formula of biotinylated DHT. **B** Biotinylated-DHT pull-down assay. Expression of the top 20 candidate proteins by mass spectrometry was presented using a heatmap after biotinylated-DHT pull-down assays in T24 cells. **C** EPPK1 was more highly expressed in five-six cancer tissues than in normal tissues, as measured by qRT‒PCR. **D** EPPK1 protein was more highly expressed in MIBC (score = 6.80 ± 3.809) than in NMIBC (4.90 ± 3.398) (*p* = 0.046) and paired paracancerous tissues (2.517 ± 2.626) (*p* < 0.001). **E** The proliferation and invasive abilities were decreased in cells with EPPK1 knockdown. **F** Genetic sequencing of EPPK1 in six male and six female patients exhibiting EPPK1 mutations was common in BLCA, five mutation sites and their positions on chromosomes can be detected in more than eight samples was shown. Scale bar = 100 μm. *p < 0.05.

To explore the possible regulatory mechanism of DHT in BLCA, we used a biotin probe to target DHT (Fig. 3A), followed by pull-down experiments. Pull-down proteins were detected by mass spectrometry, and the top 10 proteins were selected (Fig. 3B). qRT‒PCR showed that only EPPK1 mRNA levels were higher in nearly all (5/6) BLCA tissues than in the adjacent tissues (Fig. 3C). IHC detected that EPPK1 protein was more highly expressed in MIBC (score = 6.80 ± 3.809) than in NMIBC (4.90 ± 3.398) (*p* = 0.046) and paired paracancerous tissues (2.517 ± 2.626) (*p* < 0.001). (Fig. 3D). Plasmid transfection was used to knock down EPPK1 in T24 cells treated with DHT. We performed cell proliferation experiments and invasion experiments. The experimental results showed that the ability of DHT+T24-shEPPK1 to proliferate and invade was decreased compared with that of DHT+T24, whereas the ability of T24-shEPPK1 to proliferate and invade was weakened compared with that of T24 (Fig. 3E). We performed genetic sequencing of EPPK1 in six males and six females collected from the clinic, there is numeral base mutation in every sample, and the results showed the five base mutations at different sites exist simultaneously in more than eight samples of all 12 cases (Fig. 3F), but no sex differences were found in the extent and intensity of mutations. These results showed that the functions of DHT in BLCA may occur through interacting with EPPK1.

### EPPK1 regulates JUP expression in BLCA

**Fig. 4:**
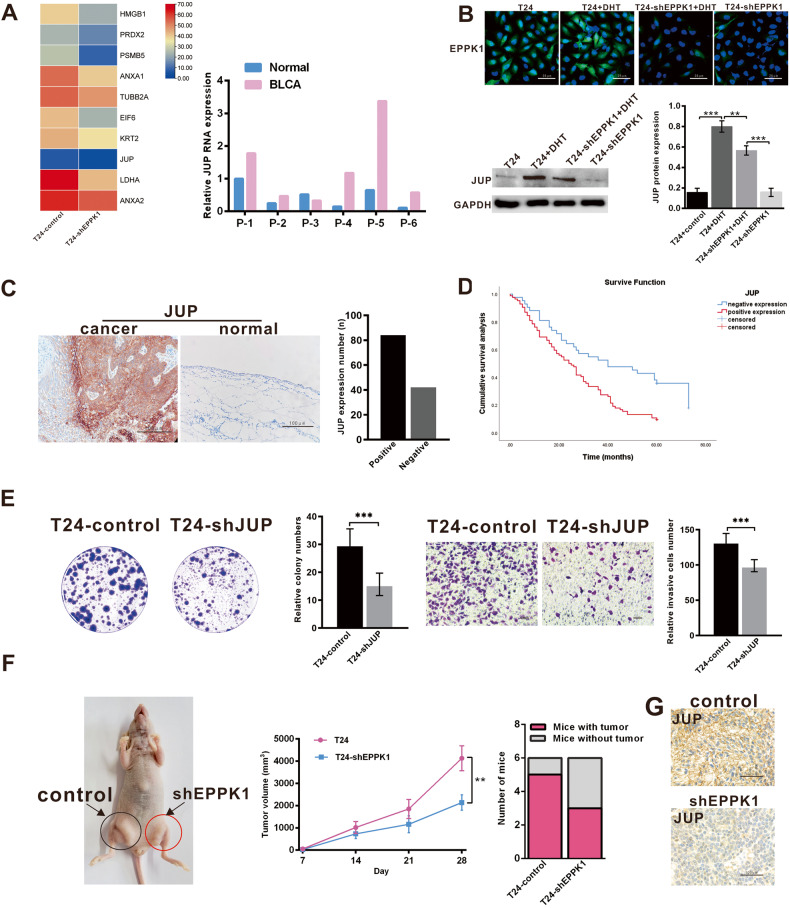
EPPK1 regulates JUP expression in bladder cancer. **A** The expression of the top 10 candidate proteins by mass spectrometry analysis is presented using a heatmap after comparison with EPPK1 knockdown T24 cells and T24 cells. The mRNA expression level of JUP in BLCA and their adjacent tissues is consistent with that of EPPK1. **B** EPPK1 and JUP protein were overexpressed in T24 cells treated with DHT and expressed at low levels in EPPK1 knockdown cells. **C** JUP expression is higher in cancer tissues than in normal tissues. **D** The survival curve of BLCA patients with JUP expression. **E** The proliferation and invasion capacity of T24 cells with JUP knockdown were decreased. **F** T24-shEPPK1 cells subcutaneously injected into BALB/c nude mice formed fewer tumours and exhibited slower growth than T24-control cells. **G** High JUP expression in T24-control cells formed tumours and decreased JUP expression in T24-shEPPK1 cells formed tumours. Scale bar = 25 μm (IF) or 100 μm (IHC). *p < 0.05, **p < 0.01, ***p < 0.001.

To further investigate the downregulated signals, the main differentially expressed protein between T24-shEPPK1 and T24-control cell lysates was identified by mass spectrometry, qRT‒PCR showed that JUP is the only gene whose mRNA expression level in BLCA and their adjacent tissues is consistent with that of EPPK1 (Fig. 4A). This result was verified by IF and WB, that is, the expression level of EPPK1 and JUP in T24+DHT group cells was increased compare with T24, whereas decreased in T24-shEPPK1, and T24-shEPPK1+DHT cells, indicating that DHT has a promoting effect on JUP and maybe regulated through EPPK1(Fig. 4B). In 126 cases BLCA patients’ tissues, there are 84 cases were positive expressed for JUP expression, and 42 were negative expressions (Fig. 4C). We subsequently performed Kaplan–Meier analyses followed by log-rank tests to assess possible associations between each expression and disease progression. Among 126 BLCA patients with a mean follow-up time of 32.952 months, the survival of patients with high JUP expression was shorter (26.761 ± 2.673) than that in the low JUP expression group (42.857 ± 12.947), log rank (Mantel‒CoxMantel-Cox) = 11.263, *p* = 0.001(Fig. 4D). Plasmid transfection was used to knock down JUP in T24 cells, and the proliferation and invasion abilities of T24-shJUP cells were decreased compared with those of the control group (Fig.4E). In a xenograft mouse model, when T24 cells stably transfected with T24-shEPPK1 or control vector were injected subcutaneously into BALB/c nude mice, the tumourigenesis rate of the control group was higher than that of the knockdown EPPK1 group (Fig. 4F). JUP was expressed at lower levels in T24-shEPPK1 group tissues.

### DHT interacts with EPPK1 to activate JUP expression through the P38/c-JUN signalling pathway

**Fig. 5:**
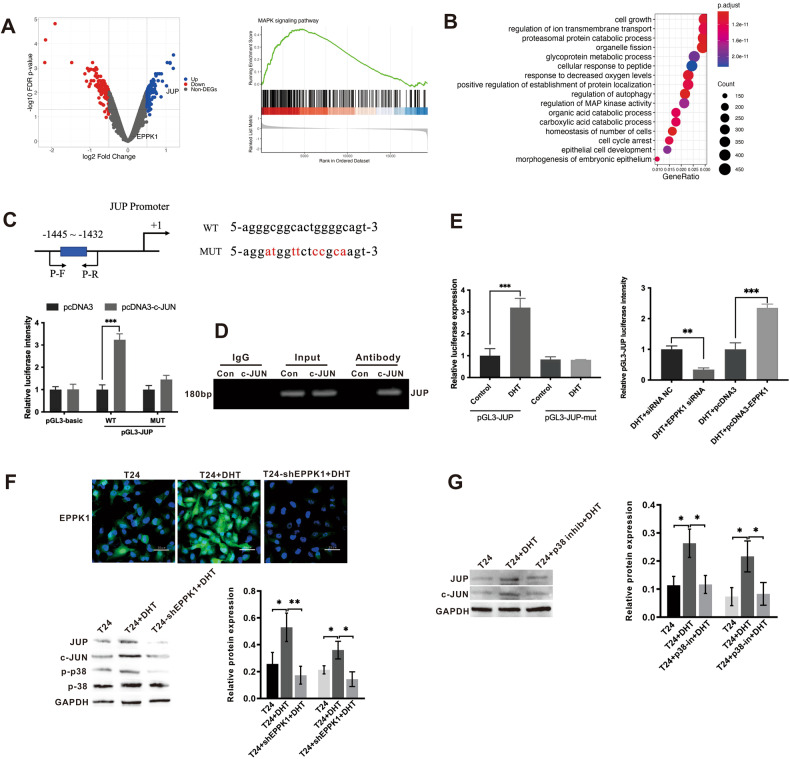
EPPK1 activates JUP expression through the p38 MAPK signalling pathway. **A** EPPK1 upstream and downstream genetic volcano map. **B** Gene Ontology enrichment analysis. **C** Luciferase activity in T24 cells cotransfected with a luciferase reporter containing c-JUN sequences with wild-type or mutated JUP binding sites and a mimic control. **D** ChIP‒qPCR was performed in T24 cells to identify c-JUN as a direct binding target of JUP. Mouse IgG was used as a negative control. **E** Luciferase activity analysis in T24 cells cotransfected with pGL3-JUP-wt/mut vectors and DHT, luciferase activity in T24 cells cotransfected with pGL3-JUP with DHT+EPPK1 or control, DHT+pcDNA3 and DHT+pcDNA3. **F** IF and Western blot analysis of the protein expression of EPPK1, JUP, c-JUN, p-p38, and p38 in T24, T24+DHT, and T24-shEPPK1+DHT cells. **G** Western blot analysis of the protein expression of JUP and c-JUN in T24, T24+DHT, and T24+p38 inhibition + DHT cells. Data are represented as the means ± S.D. of at least three independent experiments. Scale bar = 25 μm. *p < 0.05, **p < 0.01, ***p < 0.001.

We performed RNA sequencing on T24 cells treated with DHT to understand the pathway by which DHT-dependent EPPK1 activates JUP. We observed that JUP was induced after DHT treatment of T24 cells, but it did not affect the expression level of EPPK1 (Fig. 5A). Afterwards, we performed pathway analysis on DHT-treated T24 cells, and the results showed that the MAPK signalling pathway was induced, indicating that DHT changed transcription factors and JUP expression by mediating the MAPK signalling pathway (Fig. 5B). C-JUN is a transcription factor downstream of MAPK, and we predicted a potential binding site for c-JUN on the JUP promoter and mutated the potential binding site for c-JUN. Next, we verified the potential binding of c-JUN to a JUP sequence by luciferase reporter gene experiments, we demonstrated that c-JUN combined with the JUP promoter significantly increased the luciferase activity compared with using JUP alone, whereas the mutant luciferase activity was not evident (Fig. 5C). To verify whether c-JUN interacts with the endogenous promoter of JUP, ChIP-qPCR was performed in T24 cells. The results showed that c-JUN can bind the endogenous promoter of JUP (Fig. 5D). As shown in Fig. 5E after treatment of JUP and mutant JUP with DHT, the luciferase activity of JUP was increased compared with that of the control group, whereas the luciferase activity of mutant JUP did not change significantly. This result indicates that DHT can activate the JUP promoter. Adding DHT-treated EPPK1 shRNA decreased JUP promoter activity, whereas adding DHT-treated pcDNA3-EPPK1 increased JUP activity. This result indicates that DHT is dependent on EPPK1, which affects the JUP promoter. Next, we used Immunofluorescence and WB to observe changes in expression at the protein level. After DHT treatment of T24 cells, the expression levels of EPPK1, c-JUN and JUP increased, and the p38 MAPK signalling pathway was activated. However, in the DHT+T24-shEPPK1 group, the c-JUN and JUP expression levels decreased, and the p38 MAPK signalling pathway was inhibited (Fig. 5F). DHT activation of the c-JUN, JUP, and p38 MAPK signalling pathways is dependent on EPPK1. As shown in Fig. 5G, after DHT treatment of T24 cells, the expression levels of c-JUN and JUP increased compared with those in T24 cells. Adding a p38 pathway inhibitor decreased the expression levels of c-JUN and JUP. Therefore, DHT interacts with the EPPK1 protein to activate the p38 MAPK signalling pathway and then activates the downstream response transcription factor c-JUN to promote the expression of JUP.

### DHT inhibition decreases tumour formation in vivo

**Fig. 6:**
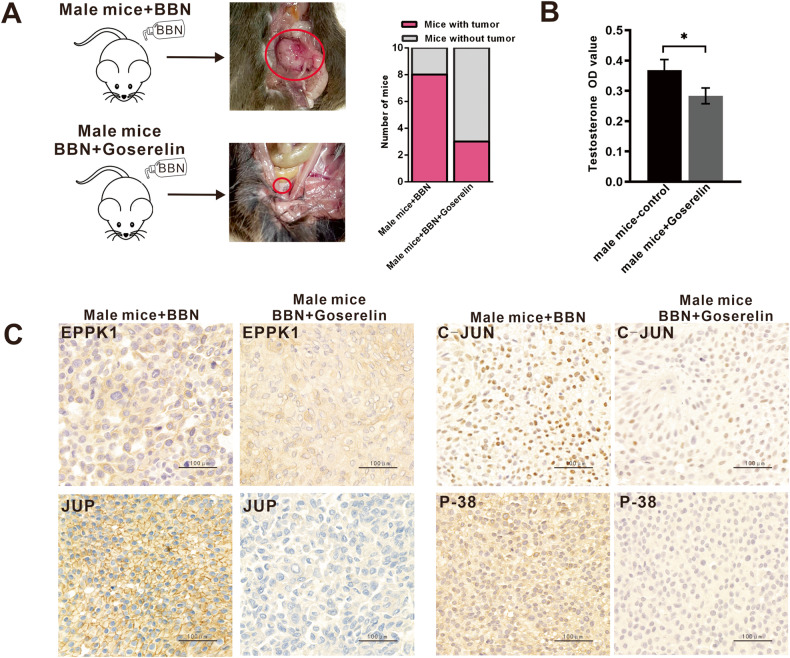
DHT inhibition decreases tumour formation in vivo. **A** The tumour formation in mice injected with goserelin was less than that in the control group. **B** Determination of testosterone content in three groups of mice by ELISA. **C** EPPK1 was highly expressed in both groups of mouse tissues, and JUP, c-JUN and p-38 were expressed at lower levels in male mice treated with BBN and goserelin than in the control group. Scale bar = 100 μm. *p < 0.05.

To further confirm the effect of DHT on BLCA, 20 mice were divided into an experimental group (10 mice) and a control group (10 mice). The mice in the experimental group were injected with goserelin. At 30 weeks of age, 80% of BBN-treated male mice developed BLCA, whereas only 30% of goserelin-injected male mice developed bladder tumours (Fig. 6A). Serum testosterone concentrations in mice were measured by ELISA, and male mice treated with BBN had higher testosterone concentrations than those injected with goserelin (Fig. 6B). Immunohistochemistry showed that EPPK1 was highly expressed in both groups of mouse tissue, and JUP, c-JUN and p38 were expressed at lower levels in male mice treated with BBN+ goserelin than in the control group (Fig. 6C). These results indicated that DHT may promote the occurrence of BLCA, which can be inhibited by androgen inhibitors. EPPK1 and p38/c-JUN/JUP signalling may be involved in this progression.

## Discussion

Androgenic action proceeds through an axis involving testosterone synthesis by the testes, its transport to target tissues, and its conversion to the active metabolite 5-dihydrotestosterone (DHT) by 5-reductase. AR signalling refers to DHT binding to AR as a ligand induces its activation to be imported into the nucleus, where AR binds to the androgen response element (ARE), which in turn acts as a transcriptional activator to promote biological functions such as tumour cell proliferation and invasion. In addition to this, incomplete forms of AR protein can be produced during the formation of certain splice isoforms of AR, which can be continuously activated independent of androgens to exert aberrant transcriptional regulation due to the lack of LBD functional domains for ligand binding [6, 28]. In this study, we demonstrated that DHT can promote BLCA cell proliferation and invasion. This carcinogen induces BLCA and metastases more frequently and more rapidly in males than in females, as observed in animal models treated with BBN, and castration can delay or reduce the development of BBN-induced BLCA. These results indicate that the occurrence of BLCA may be related to androgen. However, we found that androgen does not regulate the progression of bladder cancer through the AR pathway. We confirmed the results of Fig. 2A, B with a number of pathologists from Tianjin Medical University and Tianjin Medical University General Hospital, who similarly concluded that AR is expressed at low levels in human and mouse BLCA tissues. Based on our studies and pathologists’ clinical experience, the experts stated that the bladder is not generally considered to be an androgen-responsive organ, so we confirmed that AR expression was low in bladder cancer tissues, even in male samples. In a meta-analysis of AR protein expression studies, AR expression was found to be similar to normal uroepithelial cells in bladder cancer cells and was not significantly elevated [29]. Furthermore, a study by Sikic et al. showed no correlation between AR expression in uroepithelial cancers, including bladder cancer, and gender (*p* = 0.23) [30]. In summary, since AR expression was generally low in uroepithelial tissues, we concluded that AR was not significantly elevated in either normal or tumour tissues of the bladder, which corroborates DHT plays a role in the pathogenesis of BLCA via a non-AR pathway.

Our finding that EPPK1 can bind to DHT by performing mass spectrometry analysis was confirmed by detection in BLCA tissues. Eppk1 was originally cloned as an autoantigen in human subepidermal vesicular disease [31]. It is expressed in various progenitor, developmental, and regenerative cells, particularly in pancreatic cancer [32], indicating that it may be a regulatory factor in cancer cell development, but it has no biological or clinical implications for BLCA. During our study, we performed a preliminary functional characterisation of EPPK1 expression in BLCA. EPPK1 was found to be wildly mutated in BLCA compared with paired paracancerous tissues. The lentivirus-mediated knockdown of EPPK1 and the addition of DHT significantly inhibited the proliferation and invasion of BLCA cells in vitro, suggesting that the function of DHT in promoting cell proliferation and migration may occur through EPPK1 activation in BLCA. However, this study still has limitations. The mechanisms by which DHT regulates EPPK1 activation need to be explored in depth. In addition, EPPK1 knockdown resulted in a decrease in numeral proteins, including JUP. JUP is a cytoplasmic peripheral membrane protein that serves as a desmosome junction and adhesive component, and its canonical function is attaching cadherins to the cytoskeleton to maintain cellular structural stability [33]. Escape from the cell adhesion system is an important mechanism by which tumour cells can migrate and invade [34]. Our results confirmed that JUP played a promoting role in BLCA, cell invasion capacity was weakened after knockdown, and JUP was significantly decreased in EPPK1 knockdown cells. Therefore, cell invasion regulated by EPPK1 may occur mainly through JUP. Furthermore, JUP was considered to maintain cell adhesion in keratinocytes by inhibiting p38 MAPK; thus, the loss of JUP function leads to the activation of p38 MAPK in these cells [35].

**Fig. 7:**
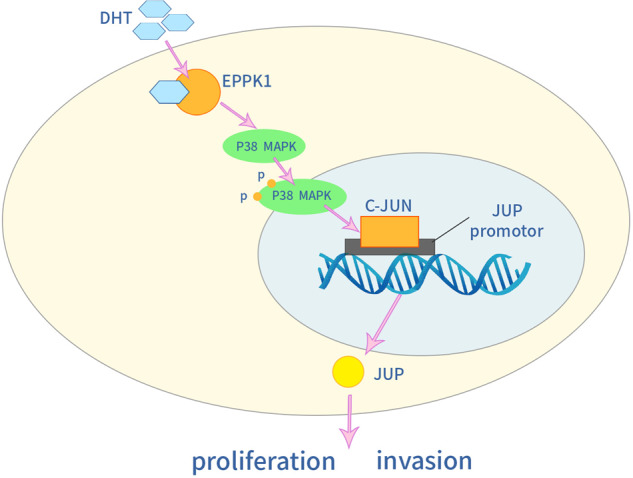
The mechanism by which DHT promotes BLCA through p38/c-Jun/JUP signalling. DHT interacts with the EPPK1 protein to activate the p38 MAPK signalling pathway and then activates the downstream response transcription factor c-JUN to promote the expression of JUP to increase cell proliferation and invasion.

MAPK responds to extracellular stimuli and mediates cellular signalling [36]. The MAPK pathway includes several key signalling components and phosphorylation events that play a role in tumour progression and metastasis. MAPKs have five major subgroups: ERK (ERK1/ERK2), c-Jun N (JNK/SAPK), p38 MAPK, ERK3/ERK4, and ERK5 [37]. JNK and p38 are induced by cellular stress and are closely related to cell death [38]. Therefore, we studied DHT-dependent EPPK1 to promote the expression of JUP through the transcription Factor c-JUN, which is a downstream response of the p38 MAPK signalling pathway, and we found that c-JUN can bind to the JUP promoter. In addition, the protein expression levels of JUP, p-p38, and p38 increased after the addition of DHT, and c-Jun, which is an important signal transduction factor activated by MAPK family members, also increased. Moreover, the expression levels of JUP, p-p38, p38, and c-Jun decreased after the knockdown of EPPK1 by adding DHT treatment. However, the expression levels of c-Jun and JUP decreased after DHT treatment and the addition of p38 inhibitors. Therefore, the occurrence of BLCA may be due to DHT-dependent EPPK1 activation of the p38 MAPK/c-JUN signalling pathway, thereby promoting the expression of JUP (Fig. 7).

Androgen-mediated non-AR pathways may include the activation of other steroid hormone receptors by DHT or its metabolites [39]. Several studies have shown that the gonadotropin-releasing hormone agonist goserelin can directly inhibit various human cancer cell lines [40–42] and androgen ablation in a dose- and time-dependent manner. Goserelin is an analogue of luteinizing hormone-releasing hormone. The long-term use of this product can inhibit the synthesis of pituitary luteinizing hormone, resulting in a decrease in male serum testosterone and female serum oestradiol. In an in vivo study, we investigated the effect of goserelin injection on BLCA cell growth. The results showed that goserelin inhibited tumour growth in mice. This result indicates that goserelin inhibits the growth of BLCA by inhibiting the synthesis and secretion of androgen. This result may provide a new perspective for clinical research.

This study helps us not only understand the molecular mechanism by which increased DHT promotes the aggressiveness and metastasis of BLCA but also elucidates the regulatory relationship among DHT, EPPK1, and JUP. DHT promotes the development and metastasis of BLCA by promoting the expression of JUP through EPPK1-dependent activation of the p38 MAPK/c-jun signalling pathway. Abnormal increases in DHT may lead to increased cancer cell invasiveness followed by refractory cancer metastasis. Therefore, androgen inhibitors such as goserelin may be an effective strategy to control BLCA progression and metastasis caused by increased DHT. Treatment outcomes for patients with advanced BLCA who receive surgery, chemotherapy or radiation remain unsatisfactory. Identifying appropriate targets and understanding the molecular basis of these pathways are important steps in developing new therapies targeting specific targets in BLCA treatment.

## Supplementary information

supplement figure1

Original Data File

aj-checklist

## Data Availability

The data that were used or analysed during the current study are available from the corresponding author upon reasonable request.

## Materials availability

The materials that were used or analysed during the current study are available from the corresponding author upon reasonable request.
