# Supplementary figures and images for: Androgen dihydrotestosterone promotes bladder cancer cell proliferation and invasion via EPPK1-mediated MAPK/JUP signalling
Source: Cell Death Dis. 2023 Jun 16;14(6):363. doi: 10.1038/s41419-023-05882-1 (PMC10275919; doi:10.1038/s41419-023-05882-1)

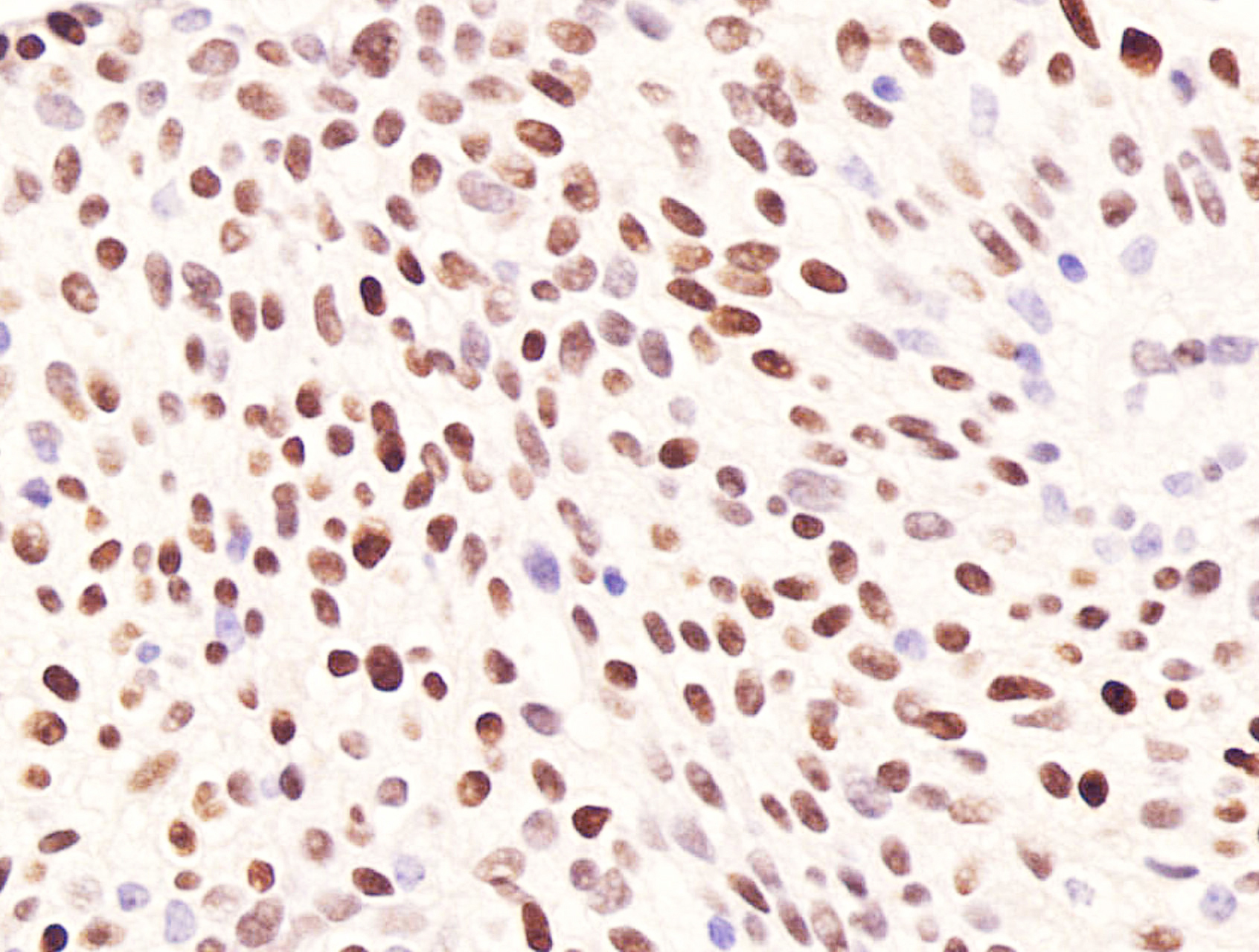

Supplement: Supplementary file 1 — supplement figure1 [file 41419_2023_5882_MOESM1_ESM.jpg]
