# Supplementary material for: Androgen dihydrotestosterone promotes bladder cancer cell proliferation and invasion via EPPK1-mediated MAPK/JUP signalling
Source: Cell Death Dis. 2023 Jun 16;14(6):363. doi: 10.1038/s41419-023-05882-1 (PMC10275919; doi:10.1038/s41419-023-05882-1)
Supplement: Supplementary file 2 — Original Data File [file 41419_2023_5882_MOESM2_ESM.pdf]

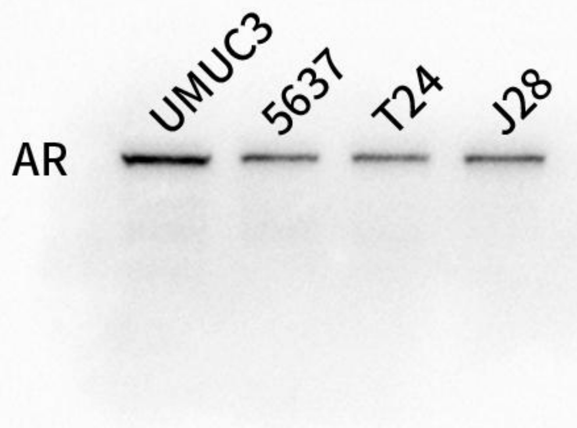
UMUC3
5637
T24
J28
AR

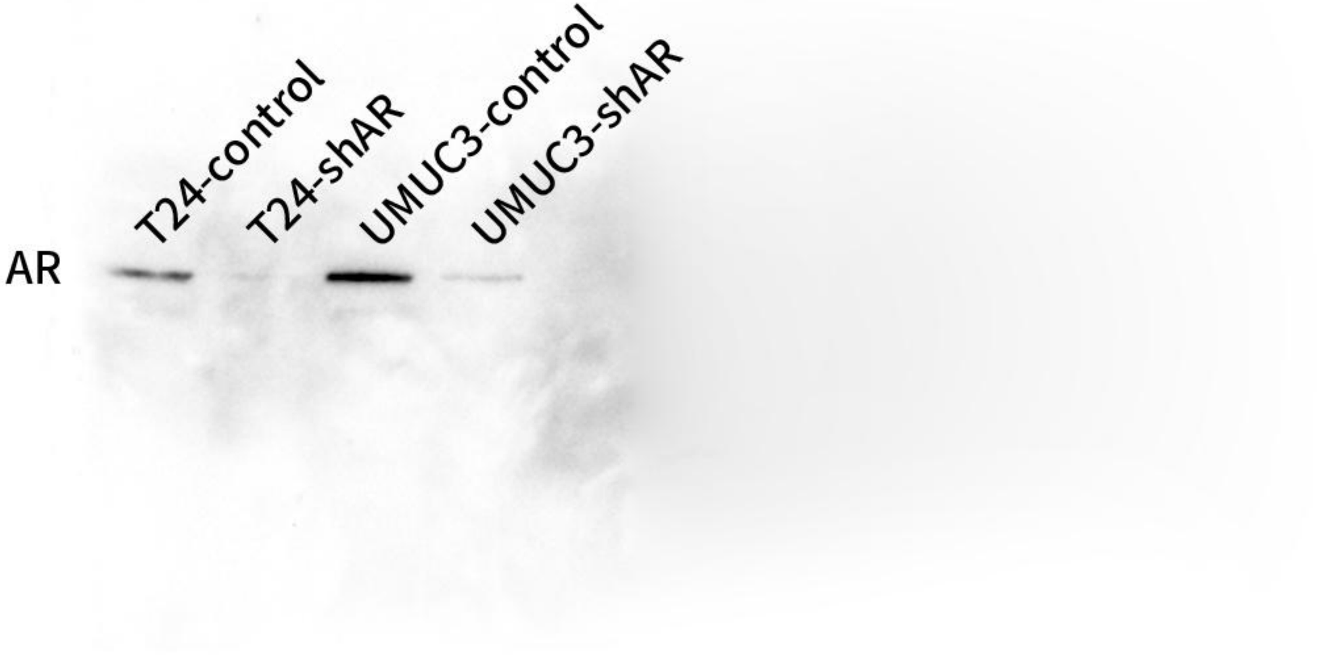
T24-control
T24-shAR
UMUC3-control
UMUC3-shAR
AR

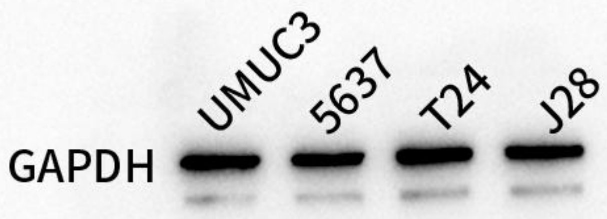
UMUC3
5637
T24
J28
GAPDH

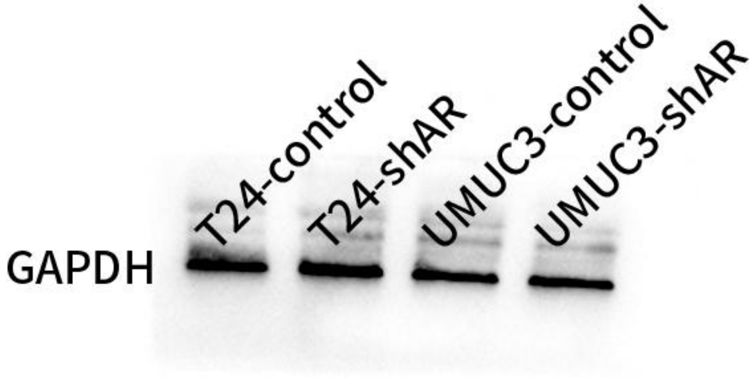
T24-control
T24-shAR
UMUC3-control
UMUC3-shAR
GAPDH

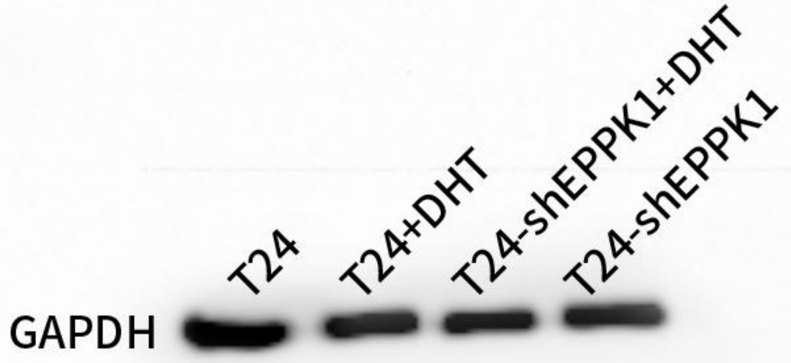
T24
T24+DHT
T24-shEPPK1+DHT
T24-shEPPK1
GAPDH

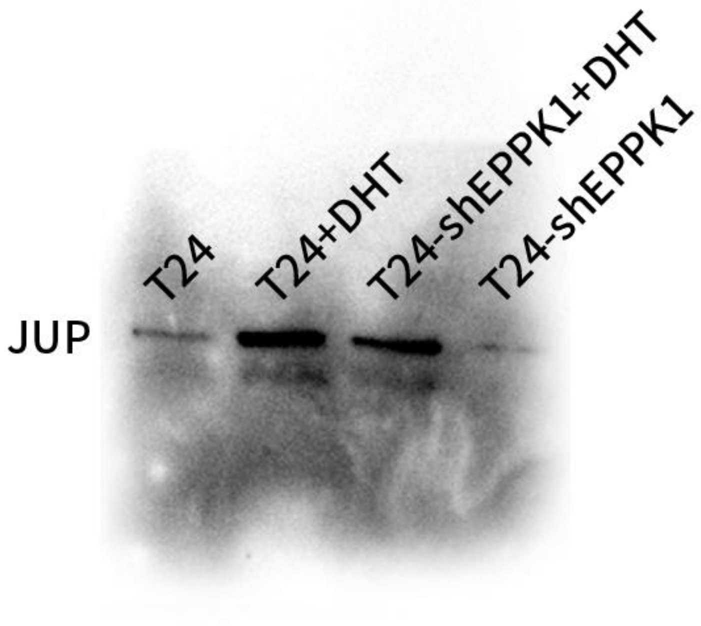
T24
T24+DHT
T24-shEPPK1+DHT
T24-shEPPK1
JUP

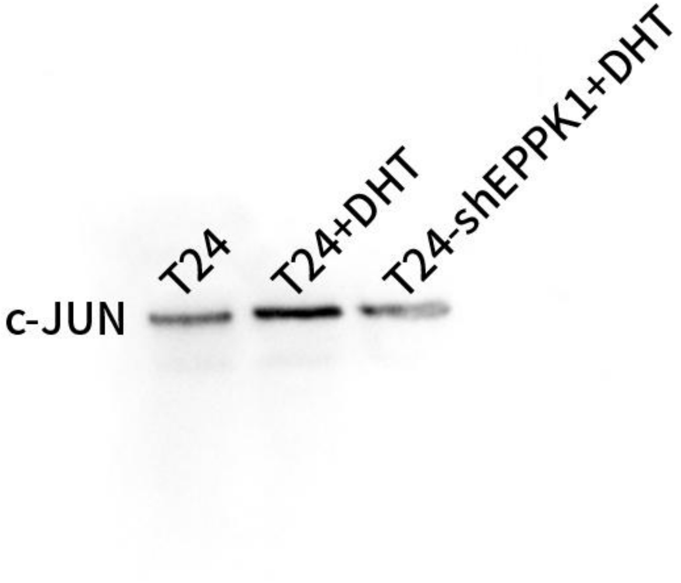
c-JUN
T24
T24+DHT
T24-shEPPK1+DHT

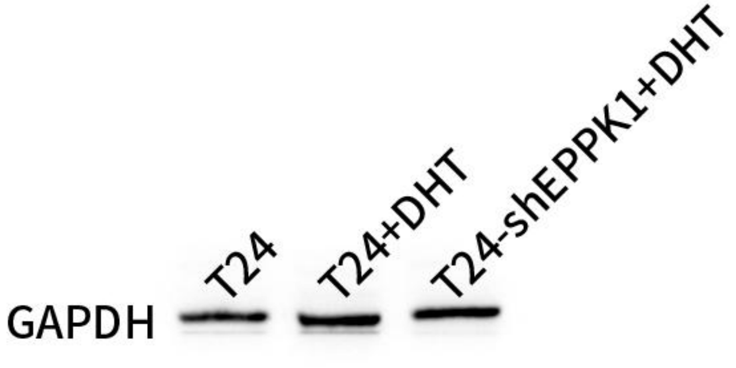
T24
T24+DHT
T24-shEPPK1+DHT
GAPDH

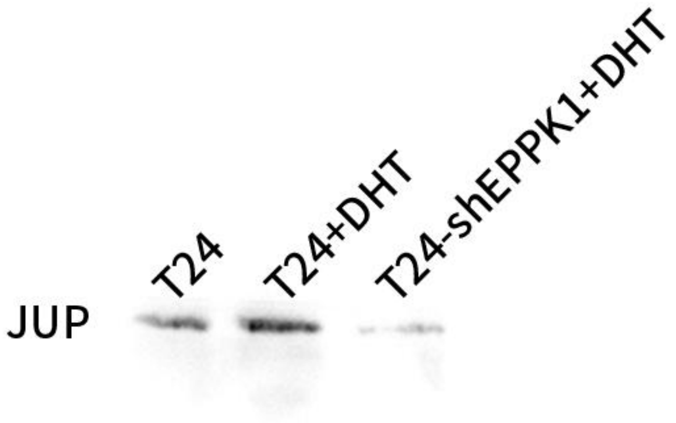
T24
T24+DHT
T24-shEPPK1+DHT
JUP

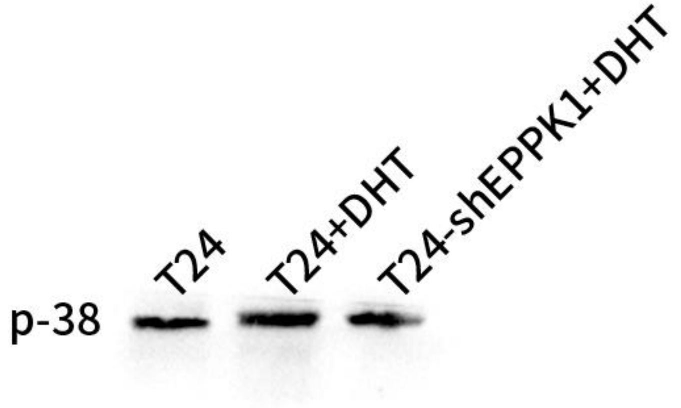
T24
T24+DHT
T24-shEPPK1+DHT
p-38

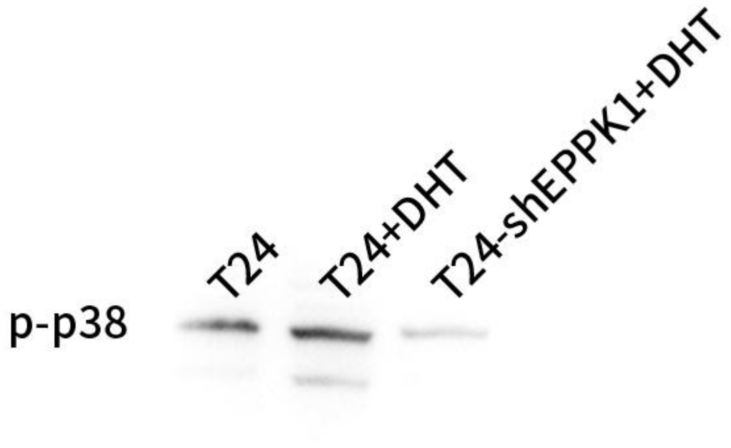

p-p38
T24
T24+DHT
T24-shEPPK1+DHT

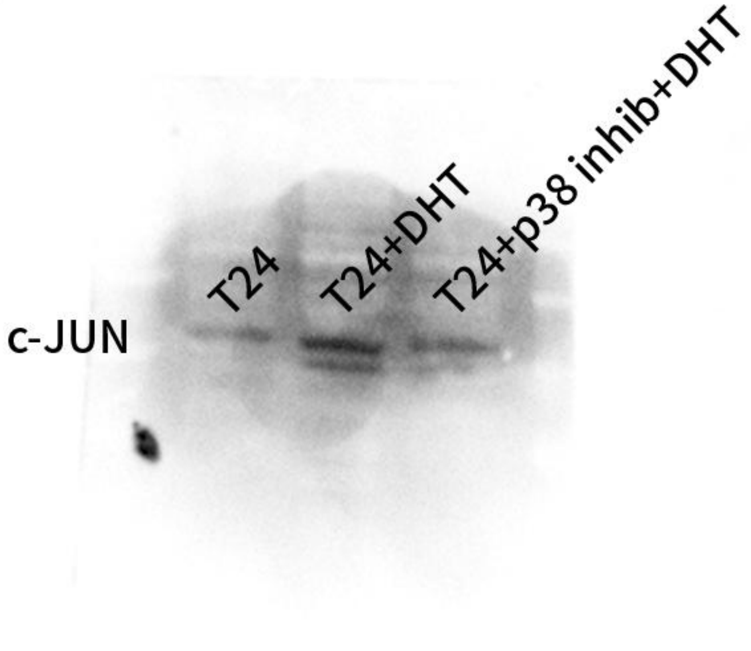
T24
T24+DHT
T24+p38 inhib+DHT
c-JUN

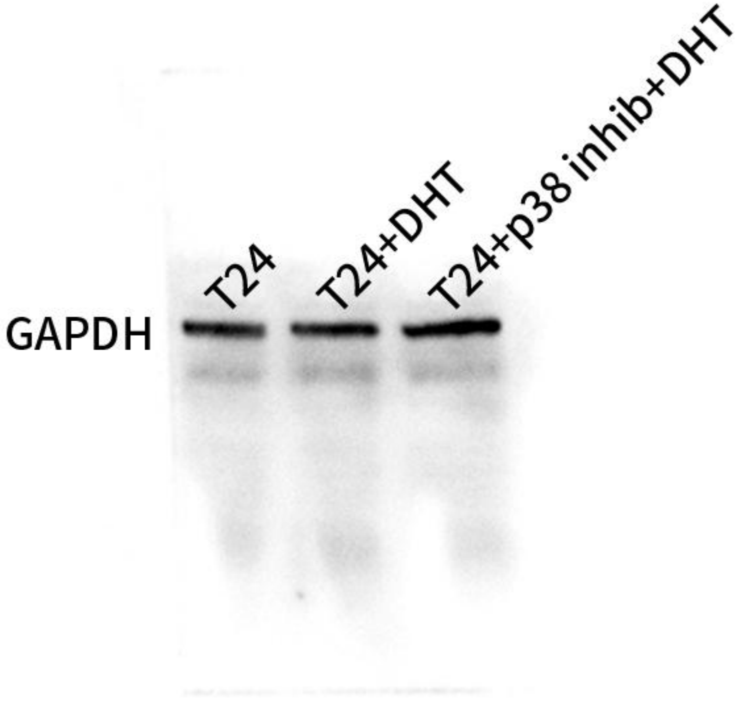
GAPDH
T24
T24+DHT
T24+p38 inhib+DHT
